# Postoperative Behavioral and Psychological Factors Associated With Recurrent Weight Gain After Metabolic and Bariatric Surgery: A Systematic Review and Meta-Analysis

**DOI:** 10.7759/cureus.117760

**Published:** 2026-10-11

**Authors:** Usman Amjad, Shaheer Ahmad, Saichandra Katragadda, Abdur R Jalali, Opeyemi S Alamu, Muhammed Z Alam Siddiqui, Mohamed Ibraheem

**Affiliations:** 1 Medicine, Al Aleem Medical College, Gulab Devi Teaching Hospital, Lahore, PAK; 2 Medicine, Antrim area Hosiptal, Antrim, GBR; 3 Medicine, Western Atlantic University School of Medicine, Freeport, BHS; 4 Education Health, SEGi university, Petaling Jaya, MYS; 5 Statistics, Federal College of Animal Health and Production Technology, Ibadan, NGA; 6 Internal Medicine, Jinnah Postgraduate Medical Center, Karachi, PAK; 7 Critical Care, Transplantation, Mayo Clinic, Phoenix, USA

**Keywords:** binge eating, depression, disinhibition, meta-analysis, metabolic and bariatric surgery, physical activity, recurrent weight gain, self-monitoring, systematic review, weight regain

## Abstract

Recurrent weight gain after metabolic and bariatric surgery (MBS) can erode long-term benefit, and many proposed correlates are modifiable. We conducted a systematic review and meta-analysis following the Preferred Reporting Items for Systematic Reviews and Meta-Analyses (PRISMA) 2020 statement. PubMed/Medical Literature Analysis and Retrieval System Online (MEDLINE), Embase, Web of Science, Scopus, and Google Scholar were searched, with citation searching, for studies of adults relating postoperative behavioral or psychological exposures to weight regain after a postoperative reference point. The databases yielded 1212 records and other sources yielded 12 (108 full texts assessed; 12 studies included); citation searching identified seven further studies, giving 19 studies (18 independent cohorts; 36 to 1278 participants). Risk of bias was appraised with Newcastle-Ottawa and Joanna Briggs Institute tools, and confidence in the evidence was rated with a structured approach informed by Grading of Recommendations Assessment, Development and Evaluation (GRADE) domains. Random-effects meta-analysis (restricted maximum likelihood, with Hartung-Knapp confidence intervals as the principal inference) was performed when at least two independent studies reported the same construct, instrument family, and metric for comparable outcomes; other evidence was synthesized narratively. Two exposures met these criteria, each pooled from the same two studies (139 participants). For eating disinhibition, the pooled standardized mean difference was 0.81, but the Hartung-Knapp 95% confidence interval (CI) included no difference (-0.40 to 2.02; I² = 0%); for depressive symptoms, it was 0.11 (Hartung-Knapp 95% CI -0.93 to 1.14; I² = 0%). In the narrative synthesis, binge eating was associated with more regain in the largest prospective cohort, and four smaller studies agreed in direction. Lower activity levels or greater sedentary behavior were associated with greater regain in most reports, although one prospective accelerometry study found no association. Confidence was low or very low, and reverse causation could not be excluded. The review was not registered. These findings identify domains that may warrant consideration in postoperative assessment, but whether screening or modification reduces recurrent weight gain remains uncertain.

## Introduction and background

Metabolic and bariatric surgery (MBS) remains the most effective treatment for severe obesity, yet weight trajectories after the lowest postoperative weight (nadir) differ widely. Some patients maintain their loss for many years, whereas others regain a clinically important share of it. The International Federation for the Surgery of Obesity and Metabolic Disorders (IFSO) now uses the term recurrent weight gain and has stressed that inconsistent terminology has made outcomes difficult to compare or pool [1]. According to an analysis of the Longitudinal Assessment of Bariatric Surgery-2 (LABS-2) cohort, the proportion of patients classified as having regained weight varies markedly with the measure applied [2], and a scoping review reached a similar conclusion for insufficient weight loss and regain [3].

Previous reviews grouped correlates of regain into dietary, psychiatric, temporal, anatomical, and genetic categories [4], or described dietary non-adherence, emotional and binge eating, depression, anxiety, and low physical activity among patients who regained weight [5]. A meta-analysis found eating psychopathology to be more common among patients with regain, whereas general psychopathology was less consistently associated [6], and a review of grazing reported an association in four of five studies [7]. However, these syntheses often combined preoperative and postoperative exposures, or merged inadequate initial weight loss with later regain [4,5]. These are different clinical problems, and whether a given behavior is linked specifically to recurrence after a nadir remains uncertain.

The objective of this systematic review and meta-analysis was to identify and synthesize empirical evidence on postoperative behavioral and psychological factors associated with recurrent weight gain after MBS in adults and to quantify these associations by random-effects meta-analysis wherever studies reported comparable estimates. Where estimates were not comparable, they were synthesized narratively.

Methods

Protocol, Reporting, and Eligibility

This systematic review and meta-analysis was conducted and reported according to the Preferred Reporting Items for Systematic Reviews and Meta-Analyses (PRISMA) 2020 statement [8]; no review protocol was registered or published. Eligible reports were original empirical studies of adults after MBS that measured a behavioral or psychological exposure after surgery and related it, with a quantitative or statistically interpretable association, to weight regain. Regain had to be measured as weight increase after the postoperative nadir or after another explicitly stated postoperative reference weight (for example, weight at 12 months); a categorical threshold was not required, so continuous regain measures were accepted. Cohort, analytical cross-sectional, case-control, and randomized designs were eligible, the latter when they reported observational exposure-regain analyses. Reports were excluded if they assessed only preoperative exposures, insufficient initial weight loss without regain, or weight loss alone; were reviews, editorials, case reports, or abstracts without extractable data; or duplicated a cohort already represented for the same exposure.

Information Sources and Search Strategy

Medical Literature Analysis and Retrieval System Online (MEDLINE) via PubMed, Embase, Web of Science Core Collection, Scopus, and Google Scholar were searched from inception without date or language limits; all sources were last searched on September 19, 2026. Three concept blocks (surgery, weight regain, and behavioral or psychological exposure) were combined with AND and terms within blocks with OR, using controlled vocabulary where available. The complete strategy for each source is given in Table 1. Twelve additional records from other sources were screened together with the database records. After inclusion decisions were made, backward citation searching of the reference lists of included studies and relevant reviews, and forward citation searching of included studies, were performed; reports found in this way were assessed against the same criteria and are shown in a separate arm of the flow diagram, outside the 1224 records.

**Table 1:** Complete search strategies and last search dates Strategies combine three concept blocks (surgery, weight regain, exposure). kw - author keywords; ti,ab - title and abstract; TS - topic field; * - truncation symbol that retrieves all word endings; exp - explode (retrieves the index term and all narrower terms); MeSH - Medical Subject Headings; MEDLINE - Medical Literature Analysis and Retrieval System Online; TITLE-ABS-KEY - title, abstract, and keyword fields

Source (platform)	Last searched	Complete search strategy
PubMed/MEDLINE (PubMed)	Sep 19, 2026	("Bariatric Surgery"[MeSH] OR bariatric OR "metabolic surgery" OR "gastric bypass" OR "sleeve gastrectomy" OR "gastric band*") AND ("weight regain" OR "weight recurrence" OR "recurrent weight gain" OR "weight recidivism") AND ("binge eating" OR "loss of control" OR grazing OR "picking and nibbling" OR "emotional eating" OR disinhibition OR craving* OR "food addiction" OR depress* OR anxiety OR "physical activity" OR sedentary OR steps OR "diet quality" OR adherence OR "self-monitoring" OR "self-weighing"). No filters.
Embase	Sep 19, 2026	('bariatric surgery'/exp OR bariatric:ti,ab,kw OR 'metabolic surgery':ti,ab,kw OR 'gastric bypass':ti,ab,kw OR 'sleeve gastrectomy':ti,ab,kw OR 'gastric band*':ti,ab,kw) AND ('weight regain':ti,ab,kw OR 'weight recurrence':ti,ab,kw OR 'recurrent weight gain':ti,ab,kw OR 'weight recidivism':ti,ab,kw) AND ('binge eating'/exp OR 'binge eating':ti,ab,kw OR 'loss of control':ti,ab,kw OR grazing:ti,ab,kw OR 'picking and nibbling':ti,ab,kw OR 'emotional eating':ti,ab,kw OR disinhibition:ti,ab,kw OR craving*:ti,ab,kw OR 'food addiction':ti,ab,kw OR depress*:ti,ab,kw OR anxiety:ti,ab,kw OR 'physical activity'/exp OR 'physical activity':ti,ab,kw OR sedentary:ti,ab,kw OR steps:ti,ab,kw OR 'diet quality':ti,ab,kw OR adherence:ti,ab,kw OR 'self-monitoring':ti,ab,kw OR 'self-weighing':ti,ab,kw). No filters.
Web of Science Core Collection (Clarivate)	Sep 19, 2026	TS=(bariatric OR "metabolic surgery" OR "gastric bypass" OR "sleeve gastrectomy" OR "gastric band*") AND TS=("weight regain" OR "weight recurrence" OR "recurrent weight gain" OR "weight recidivism") AND TS=("binge eating" OR "loss of control" OR grazing OR "picking and nibbling" OR "emotional eating" OR disinhibition OR craving* OR "food addiction" OR depress* OR anxiety OR "physical activity" OR sedentary OR steps OR "diet quality" OR adherence OR "self-monitoring" OR "self-weighing"). All editions; no filters.
Scopus (Elsevier)	Sep 19, 2026	TITLE-ABS-KEY(bariatric OR "metabolic surgery" OR "gastric bypass" OR "sleeve gastrectomy" OR "gastric band*") AND TITLE-ABS-KEY("weight regain" OR "weight recurrence" OR "recurrent weight gain" OR "weight recidivism") AND TITLE-ABS-KEY("binge eating" OR "loss of control" OR grazing OR "picking and nibbling" OR "emotional eating" OR disinhibition OR craving* OR "food addiction" OR depress* OR anxiety OR "physical activity" OR sedentary OR steps OR "diet quality" OR adherence OR "self-monitoring" OR "self-weighing"). No filters.
Google Scholar	Sep 19, 2026	Advanced search: bariatric "weight regain" OR "recurrent weight gain" OR "weight recurrence" with binge OR grazing OR disinhibition OR "physical activity" OR sedentary OR depression OR anxiety OR "self-weighing"; results screened in order of relevance; citations and patents excluded.
Citation searching	After inclusion decisions	Backward: reference lists of all included studies and of relevant systematic reviews. Forward: citing articles of each included study. Eligible reports were assessed with the same criteria and reported in a separate flow-diagram arm.

Selection, Extraction, and Overlapping Cohorts

Records were deduplicated and screened by title and abstract, followed by full-text assessment with recorded exclusion reasons. Extracted items were author, year, country, design, sample size, procedure, follow-up, exposure and instrument, regain definition, effect measure, estimate with 95% confidence interval (CI) or p-value, and principal finding. For studies eligible for meta-analysis, group means, standard deviations, and sample sizes of patients with and without regain were also extracted. Bibliographic details and key results of every included study were verified against PubMed, PubMed Central, or publisher records, and no value was imputed. Two LABS-2 reports addressed different exposure domains and were retained as non-independent reports of one cohort; they were never combined statistically.

Risk of Bias and Certainty of Evidence

Cohort studies were appraised with the selection, comparability, and outcome domains of the Newcastle-Ottawa Scale [9]; analytical cross-sectional and descriptive observational studies with the Joanna Briggs Institute (JBI) checklist for analytical cross-sectional studies; and the case-control study with the JBI checklist for case-control studies [10]. The five-year follow-up of a randomized trial, analyzed observationally, was appraised as a cohort. Certainty was rated for each exposure domain with a structured qualitative approach informed by the Grading of Recommendations Assessment, Development and Evaluation (GRADE) domains [11]; this was not a formal GRADE assessment. Evidence started at low certainty because it was observational, and was downgraded to very low when serious concerns were present for inconsistency, imprecision (wide confidence intervals, intervals compatible with both no association and an important association, or small samples with few studies), indirectness, or when most data came from studies at high risk of bias. Upgrading was not applied. Vulnerability to reverse causation was rated separately for each domain.

Effect Measures

For continuous exposures compared between patients with and without regain, the effect measure was the standardized mean difference (Hedges' g) with its 95% CI. Hedges' g was chosen because studies used instruments from the same family with different score ranges (Three-Factor Eating Questionnaire disinhibition; Beck Depression Inventory and Beck Depression Inventory-II) and because it corrects the small-sample bias of Cohen's d. All effects were coded so that positive values indicate higher scores in patients with regain. Odds ratios and adjusted mean differences were extracted as reported and displayed without pooling.

Criteria for Meta-Analysis and Data Preparation

Meta-analysis was performed only when at least two independent studies assessed the same exposure construct with the same instrument family, used comparable regain definitions, and reported the same effect metric with extractable precision. Regain definitions were considered comparable when both expressed regain as a proportion of weight lost from the postoperative nadir, even if the categorical threshold differed. Hedges' g was calculated from group means, standard deviations, and sample sizes; when only an effect size derived by the original authors from medians was available, it was used as reported and flagged. No values were imputed. Overlapping reports from the same cohort were never combined.

Meta-Analysis Model and Heterogeneity

Effect sizes were pooled with inverse-variance weighting in a random-effects model, with between-study variance (tau-squared) estimated by restricted maximum likelihood (REML). Because REML intervals are too narrow when few studies are pooled, the Hartung-Knapp-adjusted 95% CI was used as the principal basis for inference, and the conventional REML interval is shown for comparison. Heterogeneity was described with tau-squared, I-squared, and the Q test, but with two studies these statistics cannot be estimated reliably, and an I-squared of 0% was not interpreted as homogeneity. Prediction intervals were not calculated. Meta-regression and subgroup analyses were not performed because no meta-analysis included more than two studies, and both meta-analyses were therefore designated exploratory. Results are displayed as forest plots showing study-level estimates, study weights, the REML pooled estimate, and the Hartung-Knapp interval. Analyses were performed in R (R Foundation, Vienna, Austria) using the metafor package (function rma, method = "REML", test = "knha"), and figures were drawn with ggplot2.

Sensitivity Analyses, Reporting Bias, and Narrative Synthesis

Planned sensitivity analyses for the meta-analyses were exclusion of median-derived estimates and comparison with a common-effect model. Funnel plots and small-study tests were reserved for meta-analyses of at least 10 studies. As additional consistency checks, the narrative findings were re-examined after restricting the evidence to studies identified by the database search, to studies at low-to-moderate overall risk of bias, and to studies with a categorical regain threshold. All evidence that did not meet the criteria for meta-analysis was synthesized narratively by exposure domain following the Synthesis Without Meta-analysis (SWiM) guideline [12].

## Review

Study selection

The databases yielded 1212 records, and 12 additional records from other sources were screened with them (1224 in total). After 348 duplicates were removed, 876 records were screened and 760 excluded. Of 116 reports sought, eight could not be retrieved and 108 were assessed in full; 96 were excluded for wrong outcome (n = 31), wrong exposure (n = 24), wrong population (n = 8), preoperative exposure only (n = 7), wrong design (n = 9), duplicate or overlapping cohort (n = 6), insufficient extractable data (n = 5), or other unmet criteria (n = 6), leaving 12 studies. Subsequent citation searching of included studies, which is separate from the 12 other-source records, identified seven further reports; all were retrieved and met the criteria, giving 19 included studies from 18 independent cohorts (Figure 1). 

**Figure 1:**
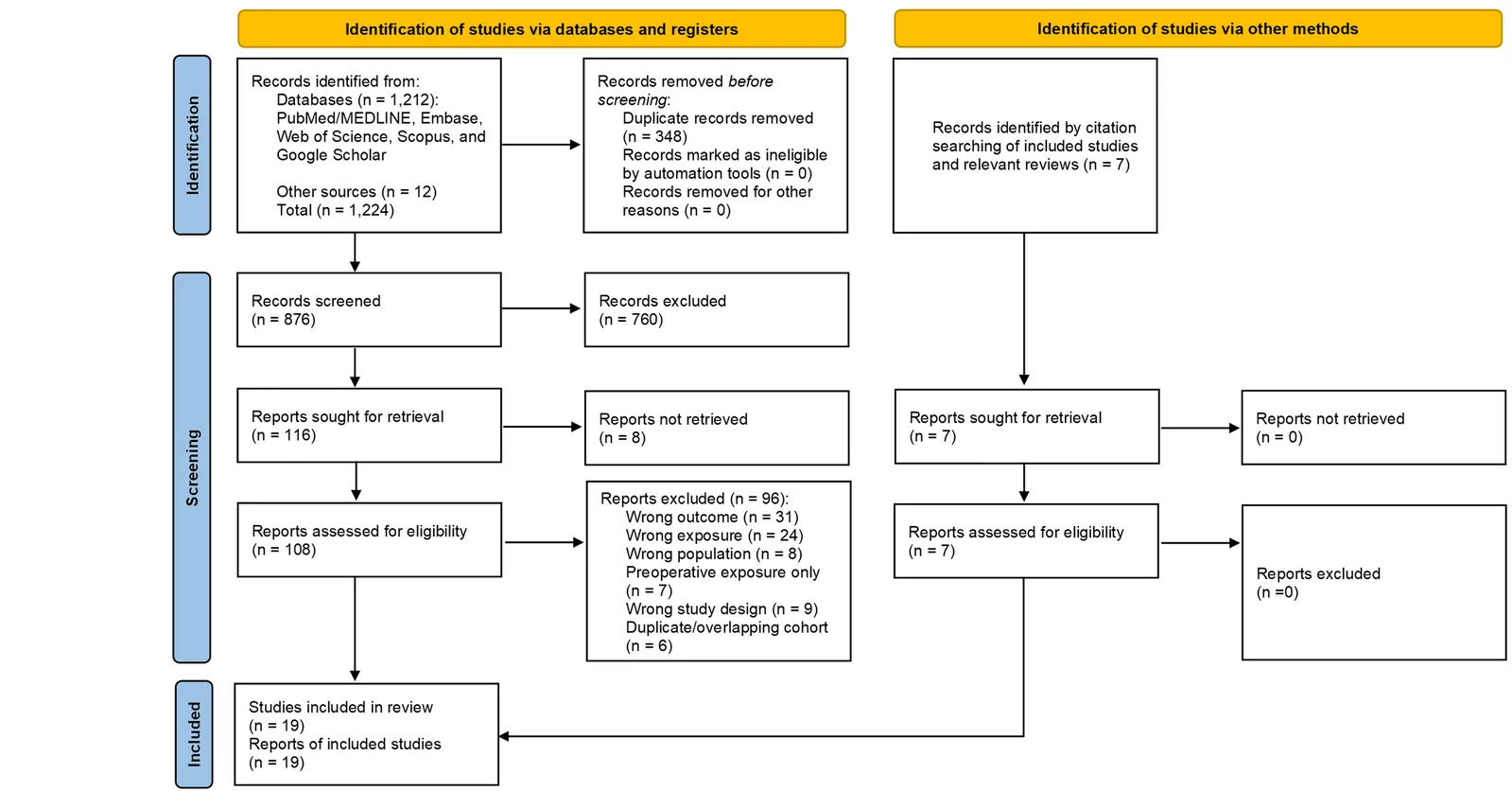
PRISMA 2020 flow diagram of study selection Nineteen studies were included in the systematic review, representing 18 independent cohorts because two reports were derived from the same LABS-2 cohort; two studies were included in the meta-analyses. PRISMA - Preferred Reporting Items for Systematic Reviews and Meta-Analyses; LABS-2 - Longitudinal Assessment of Bariatric Surgery-2

Study characteristics

The 19 studies were published between 2010 and 2025 in Brazil (seven), the United States (five), Norway (three), Portugal (two), Spain (one), and France (one) (Table 2) [13-31]. There were three prospective cohort analyses, one longitudinal study, one five-year follow-up of a randomized trial analyzed observationally, one retrospective study, and 13 cross-sectional, case-control, or descriptive studies. Samples ranged from 36 to 1278 participants (3891 across reports; 3242 after excluding the overlapping LABS-2 subsample). Roux-en-Y gastric bypass (RYGB) predominated. Regain was expressed as a fixed increase above nadir (10% or 15%), a percentage of maximum weight lost regained (thresholds from 5% to 50%), regain relative to 12-month weight loss, or a continuous or study-defined measure. Three studies analyzed weight regained after the nadir without a categorical threshold [15,16,21]; they met the eligibility criterion but were rated at higher concern for outcome measurement. Two of these studies [18,30] contributed to the meta-analyses.

**Table 2:** Characteristics of the 19 included studies †King et al. [19] and King et al. [24] derive from the same LABS-2 cohort and are treated as non-independent (19 studies, 18 independent cohorts). Effect estimates are shown with 95% confidence intervals where reported; LABS-2 estimates are adjusted differences in percentage points of maximum weight lost regained. BDI - Beck Depression Inventory; BED - binge-eating disorder; BES - Binge Eating Scale; BIS-11 - Barratt Impulsiveness Scale; DEBQ - Dutch Eating Behavior Questionnaire; EDE(-Q) - Eating Disorder Examination (Questionnaire); FCI - Food-Craving Inventory; FFQ - food frequency questionnaire; HEI - Healthy Eating Index; IPAQ - International Physical Activity Questionnaire; LABS-2 - Longitudinal Assessment of Bariatric Surgery-2; LAGB - laparoscopic adjustable gastric banding; LRYGB - laparoscopic Roux-en-Y gastric bypass; MBS - metabolic and bariatric surgery; MVPA - moderate-to-vigorous physical activity; NS - not significant; OR - odds ratio; QEWP-R - Questionnaire on Eating and Weight Patterns-Revised; RCT - randomized controlled trial; RYGB - Roux-en-Y gastric bypass; SCID-I - Structured Clinical Interview for DSM-IV Axis I Disorders; SG - sleeve gastrectomy; TFEQ - Three-Factor Eating Questionnaire; YFAS - Yale Food Addiction Scale; BDI-II - Beck Depression Inventory-II; d - Cohen's d; DSM-IV - Diagnostic and Statistical Manual of Mental Disorders, Fourth Edition; N - number of participants; Q4 - highest quartile; r - Pearson correlation coefficient; TFEQ-21 - Three-Factor Eating Questionnaire, 21-item version

Study	Country	Design	N	Procedure	Follow-up	Postoperative exposure (measure)	Regain definition	Principal finding
Odom et al. [13]	USA	Cross-sectional survey	203	RYGB	Mean 28.1 months	Food urges, well-being, alcohol/drug concerns, self-monitoring	≥15% above nadir; any regain	Food urges OR 5.10 (1.83-14.29) and alcohol/drug concerns OR 12.74 (1.73-93.80) for ≥15% regain; self-monitoring OR 0.54 (0.30-0.98) for any regain
Freire et al. [14]	Brazil	Cross-sectional	100	RYGB	Variable	Diet quality, physical activity, nutritional follow-up	>10.1% above minimum weight	Poor diet quality, sedentary lifestyle, and lack of nutritional follow-up associated with regain
Conceição et al. [15]	Portugal	Cross-sectional	313	LAGB or LRYGB	6, 12, 24 months	Binge episodes, picking and nibbling (EDE)	Weight regained after nadir, continuous	Picking and nibbling associated with regain; binge episodes uncommon
Nicolau et al. [16]	Spain	Cross-sectional	60	RYGB 50, SG 10	Mean ~46 months	Grazing (interview)	Weight regained after nadir, study-defined	Regain 72% with grazing vs 11.7% without (p < 0.0001)
da Silva et al. [17]	Brazil	Retrospective	80	RYGB	Mean 47 months	Diet quality (HEI), eating behavior, activity	≥10% above lowest weight	Adjusted OR 1.08 for time since surgery; 0.95 per HEI point
Amundsen et al. [18]	Norway	Case-control	49	RYGB	Mean ~5 years	TFEQ, DEBQ, armband activity, EDE-Q, BDI-II	>15% of weight lost regained from nadir	Higher disinhibition (7.9 vs 4.5; p = 0.02) and lower activity duration (p = 0.003); BDI-II and energy intake not different
King et al. [19]†	USA	Prospective cohort (LABS-2)	1,278	RYGB	Median 6.6 years	Eating behaviors, sedentary time, self-weighing	% of maximum weight lost regained	Binge eating +8.0, eating when full +2.9, continuous eating +1.6, sedentary Q4 +2.9, weighing less than weekly +4.2
Freire et al. [20]	Brazil	Longitudinal	96	RYGB	24 months; ~12 years	Binge eating (QEWP-R), BDI, anxiety	% of weight loss regained	Binge eating associated at both times; depression and anxiety only long term
dos Rodrigues et al. [21]	Brazil	Cross-sectional	44 women	RYGB or SG	>24 months	Physical activity, TFEQ-21	% and kg regained after nadir, continuous	Lower regain (kg) in active women (p = 0.049); activity time inversely related to % regain
Romagna et al. [22]	Brazil	Cross-sectional	90	RYGB 76, SG 14	<5 and ≥5 years	Accelerometer activity and sedentary time, IPAQ	≥20% vs <20% of weight lost regained	Low activity and more sedentary time more frequent with ≥20% regain
Bellicha et al. [23]	France	RCT follow-up, observational analysis	54	RYGB	Mean 5.7 years	Accelerometer MVPA, self-reported activity	≥10% of 12-month weight loss regained	Higher MVPA associated with lower odds of regain
King et al. [24]†	USA	Prospective cohort (LABS-2 subsample)	649	RYGB	7 years	Steps, sedentary behavior, MVPA (monitor)	% of maximum weight lost regained	Highest vs lowest step quartile 5.4% less regain (2.4-8.3); MVPA not associated
Nymo et al. [25]	Norway	Cross-sectional	75	RYGB	10-15 years	Diet (FFQ), armband activity	Regain vs no regain, study-defined	Lower activity duration with regain (p = 0.046)
Santos et al. [26]	Portugal	Retrospective and cross-sectional	84	RYGB	≥5 years	Activity, sedentary behavior, sleep (telephone questionnaires)	Weight regain, study-defined	Lower activity and more sedentary behavior associated with regain
Furtado et al. [27]	Brazil	Descriptive observational	217	RYGB or SG	Mean 5.2 years	Binge-eating disorder, depression, anxiety	Two definitions: >15% above nadir; ≥20% of maximum weight lost	Regain in 35%; associated with BED (p = 0.004), depression (p = 0.008), anxiety (p = 0.001)
Nicanor-Carreón et al. [28]	USA	Cross-sectional web survey	36 women	Metabolic surgery	2-10 years	Cravings (FCI), DEBQ, EDE-Q	% of maximum weight lost regained	Correlated with sweet cravings (r = 0.43) and EDE-Q (r = 0.38)
Sundgot-Borgen et al. [29]	Norway	Prospective cohort	79	Bariatric surgery	1 and 5 years	Accelerometer activity, steps, sedentary time	Weight recurrence at 5 years, measured weights	Changes in activity and sedentary time not associated with recurrence
Mauro et al. [30]	Brazil	Cross-sectional	90	RYGB	Median 130.5 months	SCID-I diagnoses, BES, TFEQ, BDI, BIS-11	≥20% of maximum weight lost regained	Current BED adjusted OR 6.3 (1.26-31.06); disinhibition d = 0.76; BDI d = 0.16 (NS)
Cali et al. [31]	USA	Cross-sectional	294	Mixed MBS	Variable	Binge-eating behavior (BES), food addiction (YFAS 2.0)	Cut-offs 5%-50% of weight lost	Binge eating associated at all cut-offs (≥50%: OR 3.07, 1.45-6.49); food addiction not

Risk of bias in included studies

The LABS-2 reports had the lowest risk of bias because of prospective design, measured weights, and multivariable adjustment, with objective monitoring for activity [19,24]; the Oslo cohort and the trial follow-up also used accelerometry and measured weights [23,29]. Most cross-sectional studies raised concerns about selection, self-reported nadir weight, and residual confounding, notably by time since surgery (Table 3). The two studies included in the meta-analyses were rated at moderate to high [18] and moderate [30] overall risk of bias.

**Table 3:** Risk-of-bias assessment of the included studies Ratings indicate the level of concern in each domain; outcome concern was rated high when no regain threshold was reported. JBI-ACS - Joanna Briggs Institute checklist for analytical cross-sectional studies; JBI-CC - Joanna Briggs Institute checklist for case-control studies; NOS - Newcastle-Ottawa Scale (cohort version)

Study	Tool	Selection	Exposure	Outcome	Confounding	Overall
Odom et al. [13]	JBI-ACS	High	Moderate	Moderate	Moderate	Moderate to high
Freire et al. [14]	JBI-ACS	Moderate	Moderate	Moderate	Moderate	Moderate
Conceição et al. [15]	JBI-ACS	Moderate	Low to moderate	High	High	Moderate to high
Nicolau et al. [16]	JBI-ACS	Moderate	Moderate	High	High	Moderate to high
da Silva et al. [17]	NOS	Moderate	Moderate	Moderate	Low to moderate	Moderate
Amundsen et al. [18]	JBI-CC	High	Low	Moderate	High	Moderate to high
King et al. [19]	NOS	Low	Low to moderate	Low	Low to moderate	Low to moderate
Freire et al. [20]	NOS	Moderate	Moderate	Moderate	Moderate	Moderate
dos Rodrigues et al. [21]	JBI-ACS	Moderate	Moderate	High	High	Moderate to high
Romagna et al. [22]	JBI-ACS	Moderate	Low	Moderate	High	Moderate
Bellicha et al. [23]	NOS	Moderate	Low	Low	Low to moderate	Low to moderate
King et al. [24]	NOS	Low	Low	Low	Low to moderate	Low to moderate
Nymo et al. [25]	JBI-ACS	Moderate	Low	Moderate	High	Moderate
Santos et al. [26]	JBI-ACS	Moderate	Moderate	Moderate	High	Moderate to high
Furtado et al. [27]	JBI-ACS	Moderate	Moderate	Moderate	High	Moderate to high
Nicanor-Carreón et al. [28]	JBI-ACS	High	Moderate	Moderate	High	High
Sundgot-Borgen et al. [29]	NOS	Moderate	Low	Low	Low to moderate	Low to moderate
Mauro et al. [30]	JBI-ACS	Moderate	Low	Moderate	Low to moderate	Moderate
Cali et al. [31]	JBI-ACS	Moderate	Low to moderate	Moderate	Low to moderate	Moderate

Meta-analysis

Only eating disinhibition and depressive symptoms met the criteria for meta-analysis, each with the same two independent non-randomized RYGB studies (one case-control and one cross-sectional study; 139 participants in total) [18,30]; both meta-analyses are exploratory and are displayed as forest plots in Figure 2. Eating disinhibition was higher in patients with regain in both studies (Hedges' g 0.97, 95% CI 0.27 to 1.66 [18]; 0.75, 95% CI 0.32 to 1.18 [30]). The pooled standardized mean difference was 0.81 (REML 95% CI 0.45 to 1.18), but the principal Hartung-Knapp 95% CI included no difference (-0.40 to 2.02); between-study variance was estimated at zero (tau-squared = 0; I-squared = 0%; Q test p = 0.61). For depressive symptoms, the study estimates were -0.02 (95% CI -0.69 to 0.65) [18] and 0.16 (95% CI -0.26 to 0.58) [30], and the pooled standardized mean difference was 0.11 (REML 95% CI -0.25 to 0.46; Hartung-Knapp 95% CI -0.93 to 1.14; tau-squared = 0; I-squared = 0%; Q test p = 0.66). Mauro et al. [30] contributed 72% of the weight in both analyses. With only two studies, these heterogeneity statistics cannot be read as evidence of homogeneity. Other domains combined mean differences in percentage of maximum weight lost regained, odds ratios for different thresholds, correlations, medians, and p-values that could not be converted without unsupported assumptions; meta-analysis decisions for each domain are summarized in Table 4. Adjusted estimates reported with 95% CIs are shown unpooled in Figure 3.

**Figure 2:**
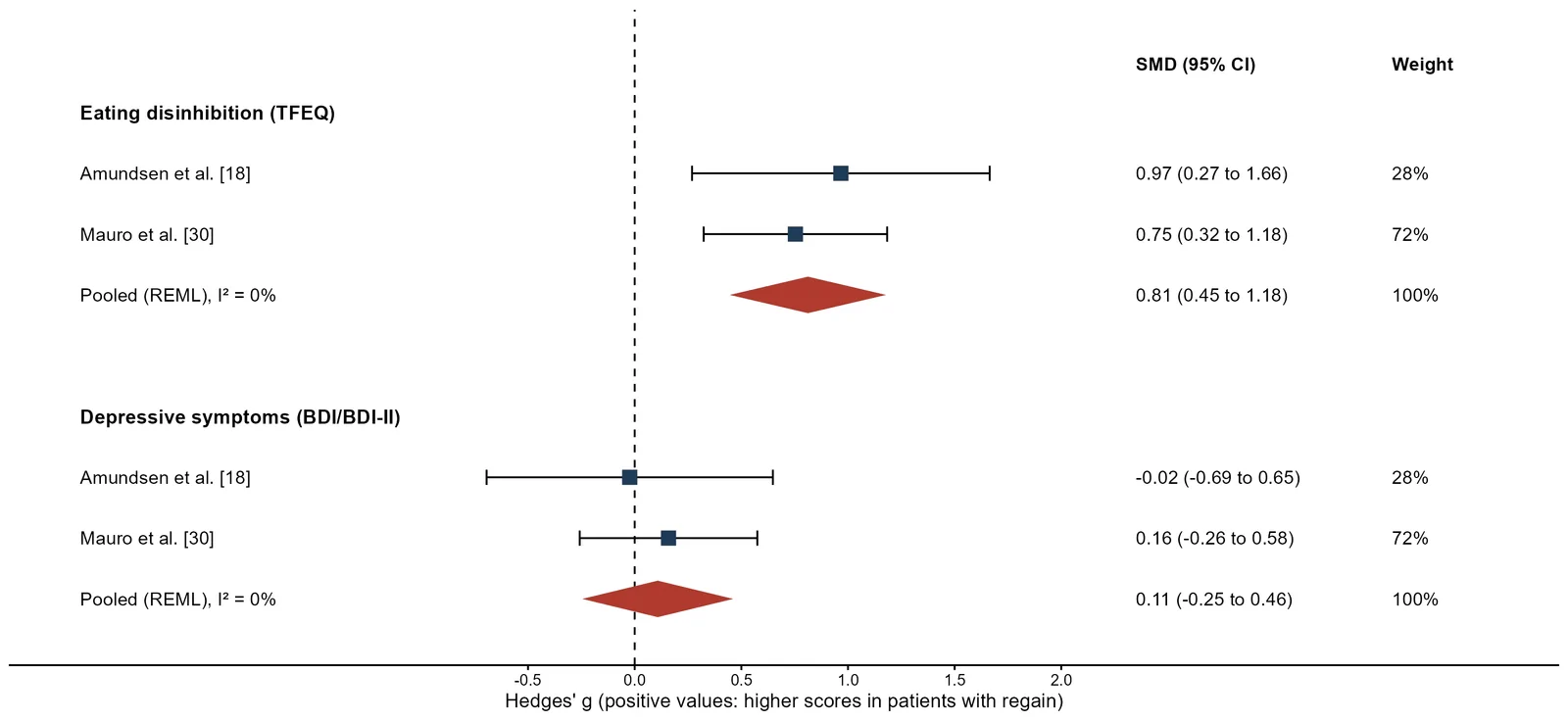
Forest plots of exploratory random-effects meta-analyses of eating disinhibition and depressive symptoms in patients with versus without weight regain Standardized mean differences (Hedges' g) above zero indicate higher scores in patients with regain. Both studies enrolled patients after Roux-en-Y gastric bypass and defined regain as a percentage of weight lost regained from nadir (>15% and ≥20%). Pooled estimates are unadjusted. Squares show study estimates sized by weight; the diamond shows the REML pooled estimate and its 95% CI; the bar below it shows the Hartung-Knapp 95% CI, which is the principal basis for inference. With two studies, I-squared cannot be interpreted as evidence of homogeneity. REML - restricted maximum likelihood; CI - confidence interval; SMD - standardized mean difference; TFEQ - Three-Factor Eating Questionnaire; BDI - Beck Depression Inventory; BDI-II - Beck Depression Inventory-II; I² - proportion of variability attributable to between-study heterogeneity; τ² - between-study variance

Sensitivity analyses and reporting bias

Because between-study variance was estimated at zero, the common-effect model gave the same estimates as the random-effects model (disinhibition 0.81, 95% CI 0.45 to 1.18; depressive symptoms 0.11, 95% CI -0.25 to 0.46). Excluding the median-derived disinhibition estimate [30] left a single study, so no pooled estimate could be recalculated; that study still showed higher disinhibition in patients with regain (g 0.97, 95% CI 0.27 to 1.66) [18]. Leave-one-out analysis was not informative with two studies. Funnel plots and small-study tests were not performed because neither meta-analysis approached 10 studies [32], so publication bias could not be assessed.

Narrative synthesis by exposure domain

Evidence that did not meet the criteria for pooling is summarized below by exposure domain, and adjusted estimates with 95% CIs are displayed without pooling in Figure 3.

**Figure 3:**
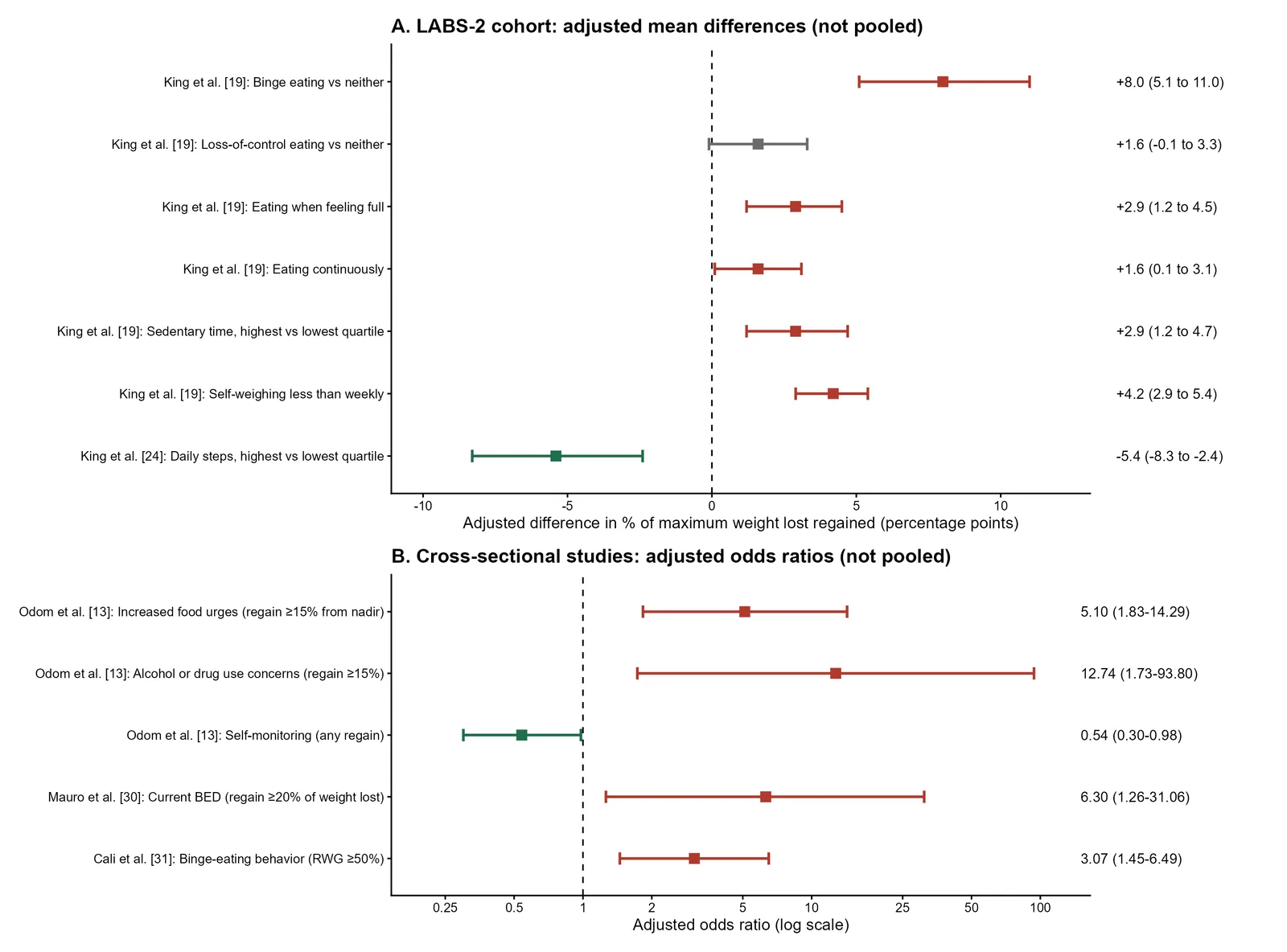
Adjusted effect estimates for postoperative exposures and recurrent weight gain, displayed without pooling Panel A shows adjusted mean differences from two reports of the LABS-2 cohort. Panel B shows adjusted odds ratios from cross-sectional studies with different exposures and thresholds. Red markers indicate more regain, green indicates less regain, and grey intervals indicate crossing the null. These estimates were not meta-analyzed because they came from one cohort (Panel A) or from different exposures and thresholds (Panel B). Values shown to the right of each panel are point estimates with 95% CIs. LABS-2 - Longitudinal Assessment of Bariatric Surgery-2; BED - binge-eating disorder; RWG - recurrent weight gain; CI - confidence interval.

Binge eating and loss-of-control eating

Binge eating was examined in six studies and was associated with more regain in five. In LABS-2, postoperative binge eating was associated with 8.0 additional percentage points of maximum weight lost regained (95% CI 5.1-11.0), whereas loss-of-control eating without bingeing had an imprecise estimate (1.6; 95% CI -0.1 to 3.3) [19]. Freire et al. found associations at 24 months (p = 0.002) and after about 12 years (p = 0.001) [20], Furtado et al. reported an association with binge-eating disorder (p = 0.004) [27], and Mauro et al. found that current binge-eating disorder remained associated with regain of at least 20% of maximum weight lost after adjustment (odds ratio (OR) 6.3; 95% CI 1.26-31.06) [30]. Cali et al. reported adjusted associations at every cut-off, highest at 50% or more (OR 3.07; 95% CI 1.45-6.49) [31]. The exception, Conceição et al., found binge episodes uncommon in the first two postoperative years, with picking and nibbling rather than bingeing associated with regain [15].

Disinhibition, grazing, and unstructured eating

Nicolau et al. reported regain in 72% of patients who grazed compared with 11.7% of those who did not (p < 0.0001) [16], and Conceição et al. identified picking and nibbling as the behavior associated with regain [15]. In LABS-2, eating when feeling full (2.9 percentage points; 95% CI 1.2-4.5) and eating continuously (1.6; 95% CI 0.1-3.1) were independently associated with greater regain [19]. Two RYGB samples measured eating disinhibition with the Three-Factor Eating Questionnaire against regain expressed as a percentage of weight lost: disinhibition was higher in patients with regain in Amundsen et al. (7.9 ± 3.3 versus 4.5 ± 4.0; p = 0.02) [18] and in Mauro et al. (Cohen's d = 0.76; p = 0.001) [30]. Although the regain thresholds differed (>15% versus ≥20% of weight lost), both studies expressed regain as a proportion of weight lost from nadir and used the same instrument, so they were pooled in the exploratory meta-analysis reported above (Figure 2).

Physical activity and sedentary behavior

Ten reports from nine cohorts assessed activity. In LABS-2, the highest quartile of sedentary time was associated with 2.9 more percentage points of maximum weight lost regained (95% CI 1.2-4.7) [19], and the highest step-count quartile with 5.4% less (95% CI 2.4-8.3), whereas moderate-to-vigorous physical activity (MVPA) was not associated [24]. Armband-measured activity duration was lower in patients with significant regain in Amundsen et al. (p = 0.003) [18] and Nymo et al. (p = 0.046) [25], and Romagna et al. found low activity and more sedentary time more often among patients regaining at least 20% of weight lost [22]. In the five-year follow-up of a randomized trial, higher accelerometer-measured MVPA was associated with lower odds of regaining at least 10% of 12-month weight loss [23]. Lower activity was also associated with regain in three further samples [14,21,26]. In contrast, in a prospective cohort with accelerometry at one and five years, changes in activity and sedentary time were not associated with weight recurrence [29]. Pooling was not possible because activity was summarized as medians, quartile contrasts, odds ratios, or p values.

Self-monitoring, diet quality, and cravings

Self-monitoring was associated with lower odds of any regain (OR 0.54; 95% CI 0.30-0.98) [13], and weighing less than weekly with 4.2 additional percentage points of maximum weight lost regained (95% CI 2.9-5.4) [19]. Increased food urges were associated with regain of at least 15% from nadir (OR 5.10; 95% CI 1.83-14.29) [13]. Poor diet quality was linked with regain in two Brazilian RYGB samples, one reporting an adjusted OR of 0.95 per Healthy Eating Index point [14,17], and sweet cravings (r = 0.43) and Eating Disorder Examination Questionnaire global scores (r = 0.38) correlated with regain in 36 women [28]. However, energy intake did not differ between patients with and without significant regain in Amundsen et al. [18].

Depression, anxiety, and food addiction

Freire et al. found no association with depressive or anxiety symptoms at 24 months, but associations at long-term follow-up (p = 0.029 and p = 0.001) [20]; Furtado et al. reported associations with depression (p = 0.008) and anxiety (p = 0.001) [27]; and LABS-2 listed more depressive symptoms among characteristics associated with regain [19]. By contrast, Beck Depression Inventory scores did not differ by regain status in Amundsen et al. [18] or Mauro et al. [30]; in the exploratory meta-analysis of these two studies, the pooled standardized mean difference was small and imprecise (0.11; Hartung-Knapp 95% CI -0.93 to 1.14) (Figure 2), which indicates no clear difference but cannot establish the absence of an association. Food addiction was not associated with regain at any cut-off in the only study that assessed it, whereas binge-eating behavior in the same sample was [31].

Alcohol use, impulsivity, and other psychopathology

Two studies examined substance use. Odom et al. reported that concerns about alcohol or drug use were associated with regain of at least 15% from nadir (OR 12.74; 95% CI 1.73-93.80), with a very wide interval [13]. Mauro et al. found lifetime alcohol abuse or dependence more often among patients with regain (20% versus 5%; p = 0.03), together with lifetime obsessive-compulsive disorder and bulimia nervosa, but none of these remained associated after adjustment, whereas current binge-eating disorder did [30]. The same study reported higher non-planning impulsivity in patients with regain (d = 0.69; p = 0.0001) [30]. Severely decreased postoperative well-being was also associated with significant regain in Odom et al. (OR 21.5; 95% CI 2.50-183.10) [13].

Consistency checks of the narrative synthesis

Restricting the evidence to the 12 studies from the database search did not change the direction of the binge-eating or activity findings: binge eating remained associated with regain in four of five studies [15,19,20,27,31] and lower activity in all four activity reports [14,19,21,24]. However, both pooled analyses and the null findings for depressive symptoms came from studies identified by citation searching [18,30], so the conclusion that depression is inconsistently associated with regain depends on these additions. Restricting to studies at low-to-moderate overall risk of bias left binge eating supported by LABS-2 alone and activity supported by three of four reports, with the Oslo cohort null [19,23,24,29]. Excluding the three studies without a categorical threshold [15,16,21] removed most direct evidence on grazing and picking and nibbling.

Certainty of evidence

Certainty was low for binge eating, physical activity and sedentary time, and self-monitoring, and very low for disinhibition and grazing, diet quality and cravings, depression and anxiety, alcohol or substance use, and food addiction (Table 4).

**Table 4:** Summary of evidence, meta-analysis decisions, and confidence by exposure domain Participants are summed across contributing reports; the overlapping LABS-2 activity subsample is not counted twice. Meta-analysis required at least two independent studies with the same construct, instrument family, metric, and comparable regain definition. Certainty was rated with a structured approach informed by GRADE domains, starting at low for observational evidence. BED - binge-eating disorder; EDE-Q - Eating Disorder Examination Questionnaire; GRADE - Grading of Recommendations Assessment, Development and Evaluation; HK - Hartung-Knapp; REML - restricted maximum likelihood; LABS-2 - Longitudinal Assessment of Bariatric Surgery-2; OR - odds ratio; SMD - standardized mean difference

Exposure domain	Studies (participants)	Direction	Meta-analysis decision	Certainty (main reasons)	Reverse-causation vulnerability
Binge eating / BED	6 (2288) [15,19,20,27,30,31]	More regain in 5 of 6	Not pooled: one mean difference, two ORs for different thresholds, p-values	Low (consistent; prospective data; heterogeneous definitions)	Moderate
Disinhibition, grazing, unstructured eating	5 (1790) [15,16,18,19,30]	More regain in all 5	Meta-analyzed (2 studies, exploratory): SMD 0.81 (REML 95% CI 0.45 to 1.18; HK 95% CI -0.40 to 2.02); others not poolable	Very low (imprecision; small cross-sectional samples; varied constructs)	High
Physical activity / sedentary time	10 reports, 9 cohorts (1853 unique) [14,18,19,21-26,29]	Less activity, more regain in 9 of 10; one prospective null	Not pooled: medians, quartile contrasts, ORs, p-values	Low (mostly consistent; objective measures; one null prospective study)	High in cross-sectional data
Self-monitoring / self-weighing	2 (1481) [13,19]	Monitoring, less regain	Not pooled: OR vs mean difference, different outcomes	Low (consistent; large sample)	High
Diet quality, food urges, cravings, EDE-Q	5 (468) [13,14,17,18,28]	More regain in 4; energy intake null in 1	Not pooled: incompatible metrics	Very low (inconsistency; small samples)	High
Depression / anxiety	5 (1730) [18-20,27,30]	Associated in 3; null in 2	Depressive symptoms meta-analyzed (two studies, exploratory): SMD 0.11 (REML 95% CI -0.25 to 0.46; HK 95% CI -0.93 to 1.14)	Very low (inconsistency; timing-dependent)	High
Alcohol or substance use	2 (293) [13,30]	More regain in both (unadjusted in 1)	Not pooled: OR vs proportions	Very low (imprecision; attenuated after adjustment)	Moderate
Food addiction	1 (294) [31]	No association	Single study	Very low (single study; imprecision)	Moderate

Discussion

This systematic review and meta-analysis of 19 studies from 18 cohorts found that the evidence on postoperative behavioral and psychological correlates of recurrent weight gain is consistent in direction for some domains but rarely comparable enough to pool. Firstly, binge eating showed the most consistent direction of association among the included behavioral exposures, supported by five of six studies across countries, procedures, instruments, and definitions, including the largest prospective cohort [19,20,27,30,31]. The one discordant study assessed patients in the first two postoperative years, when binge episodes are uncommon [15]. This timing is relevant because LABS-2 data show loss-of-control and binge eating declining early after surgery and then increasing, with many new cases later [33]. The finding is consistent with a meta-analysis linking eating psychopathology to regain [6].

Eating disinhibition, a broader tendency to overeat in response to external or emotional cues, was the only behavioral exposure that met the criteria for meta-analysis, and the moderate-to-large pooled difference agrees with the grazing and unstructured-eating findings [7,15,16,19]. However, the meta-analysis contained two small non-randomized samples (one case-control and one cross-sectional), and the Hartung-Knapp interval crossed zero, so the estimate should be viewed as exploratory. One effect size was derived by the original authors from median differences [30], which adds uncertainty to its comparability with a mean-based estimate.

Physical activity findings were directionally consistent in nine of 10 reports but not uniform. The prospective Oslo cohort, which related changes in accelerometer-measured activity between one and five years to weight recurrence, found no association [29], whereas LABS-2 found associations for steps and sedentary time but not MVPA [19,24]. Differences in exposure definition (level versus change), follow-up length, and statistical power may explain this discrepancy. According to the combined evidence, total daily movement may be more closely related to regain than structured exercise, but this remains a hypothesis.

Procedure and timing also shape interpretation. Most prospective and meta-analyzed data came from RYGB, whereas sleeve gastrectomy appeared only in mixed samples, and follow-up ranged from six months to more than a decade. Behaviors measured early after surgery may not represent those present when regain accelerates, and single time-point assessments many years after surgery cannot show whether a behavior preceded regain. Accordingly, the associations summarized here are best regarded as markers present during the period of regain rather than established predictors identified before it begins.

Alcohol use and impulsivity deserve attention because they point to a broader impulsivity-compulsivity profile rather than to eating behavior alone. However, the associations for substance use were imprecise in the survey-based study [13] and did not survive adjustment in the interview-based study [30], and alcohol intake after RYGB may itself change with weight status. These domains should be assessed in future prospective studies rather than treated as established correlates.

Temporality and reverse causation

Reverse causation is a central threat to interpretation. Whether weight regain leads patients to stop weighing themselves, reduces their mobility, lowers their mood, or triggers disinhibited eating cannot be separated from the opposite direction in cross-sectional data, and 13 of the 19 studies measured exposure and outcome at the same time. The null meta-analytic result for depressive symptoms and the time-dependent associations in Freire et al. [20] fit a model in which mood changes may follow rather than precede regain. Time since surgery further confounds most associations, as it predicted regain independently in da Silva et al. and Mauro et al. [17,30]. Accordingly, Table 4 rates reverse-causation vulnerability as high for self-monitoring, depression, anxiety, and cross-sectional activity data, and as moderate for binge eating, where prospective data are available.

Comparison with previous reviews

This review extends earlier syntheses [4,5] by restricting inclusion to postoperative exposures and to regain measured from a postoperative reference weight, which separates recurrence from inadequate initial response, and by testing poolability explicitly. The resulting evidence base supports only two small exploratory meta-analyses, which reflects the inconsistent terminology highlighted by IFSO and others [1,3].

Implications for measurement and reporting

The limited poolability reflects avoidable reporting choices as much as true heterogeneity. Binge eating was assessed with the Binge Eating Scale, the Questionnaire on Eating and Weight Patterns-Revised, the Eating Disorder Examination, and structured diagnostic interviews, and activity was reported as quartile contrasts, medians, changes, or odds ratios. Instead, reporting group means with standard deviations for patients above and below a standard regain threshold, together with adjusted estimates for continuous regain, would allow future syntheses to pool results across studies. Distinguishing level of activity from change in activity is also important, because the two prospective accelerometer studies addressed different questions and reached different conclusions [24,29].

Clinical implications

These findings identify behavioral and psychological domains, particularly binge eating, disinhibited or unstructured eating, and low daily movement, that may warrant consideration in postoperative assessment. Because disordered eating can emerge years after surgery, repeated rather than single assessment is likely to be more informative. However, whether targeted screening or modification of any of these factors reduces recurrent weight gain remains uncertain and requires interventional evidence.

Strengths

This review restricted exposures to the postoperative period and outcomes to regain from a defined postoperative reference weight, verified all extracted estimates against primary sources, handled overlapping cohorts explicitly, extended the database search with systematic citation searching, and applied explicit criteria for meta-analysis rather than combining incompatible metrics. The meta-analyses used the Hartung-Knapp adjustment, which is suited to syntheses of very few studies, and confidence and reverse-causation vulnerability were rated transparently for each domain.

Limitations

The evidence was mostly cross-sectional, heterogeneous in definitions and instruments, and at moderate to high risk of bias. Seven of the 19 included studies (37%) were identified by citation searching rather than the database search, which indicates that the database strategy was not fully sensitive; although citation searching was systematic and the full strategies are reported, other eligible studies may have been missed. The protocol was not registered. Three studies lacked a categorical regain threshold [15,16,21]; excluding them would leave grazing supported only by LABS-2 eating-when-full and continuous-eating data. Each meta-analysis included only two small, unadjusted studies with different regain thresholds, so both are exploratory; heterogeneity could not be estimated reliably, one estimate was derived from medians, the planned sensitivity analyses could not produce an alternative pooled estimate, and publication bias could not be assessed.

Research implications

Future studies should adopt IFSO terminology, report regain from a measured nadir as both a continuous measure and standard thresholds [1,2], measure exposures repeatedly with validated instruments, adjust for time since surgery, and report means with standard deviations or adjusted estimates with 95% CIs to enable pooling. Prospective sleeve gastrectomy cohorts and randomized trials of targeted behavioral interventions are the main gaps.

## Conclusions

In this systematic review and meta-analysis of 19 studies, postoperative binge eating showed the most consistent direction of association with recurrent weight gain after MBS. Two exploratory two-study meta-analyses did not establish an association with eating disinhibition (SMD 0.81; Hartung-Knapp 95% CI -0.40 to 2.02) or with depressive symptoms (SMD 0.11; Hartung-Knapp 95% CI -0.93 to 1.14). Lower physical activity or more sedentary time accompanied regain in most but not all studies. Less frequent self-monitoring and poorer diet quality were linked with more regain in a small number of studies; depressive symptoms showed inconsistent associations, and food addiction showed none. Confidence in the evidence was low or very low. In conclusion, these findings identify behavioral and psychological domains that may warrant consideration in postoperative assessment, although whether targeted screening or modification improves recurrent weight gain remains uncertain.
